# Clinicopathological and prognostic significance of programmed cell death ligand 1 expression in patients diagnosed with breast cancer: meta-analysis

**DOI:** 10.1093/bjs/znab103

**Published:** 2021-05-08

**Authors:** M G Davey, É J Ryan, M S Davey, A J Lowery, N Miller, M J Kerin

**Affiliations:** 1 Lambe Institute for Translational Research, National University of Ireland, Galway, Ireland; 2 Department of Surgery, Galway University Hospitals, Galway, Ireland; 3 Department of Surgery, Royal College of Surgeons in Ireland, Dublin, Ireland

## Abstract

**Background:**

Uncertainty exists regarding the clinical relevance of programmed cell death ligand 1 (PD-L1) expression in breast cancer.

**Methods:**

A systematic review was performed in accordance with PRISMA guidelines. Observational studies that compared high *versus* low expression of PD-L1 on breast cancer cells were identified. Log hazard ratios (HRs) for disease-free and overall survival and their standard errors were calculated from Kaplan–Meier curves or Cox regression analyses, and pooled using the inverse-variance method. Dichotomous variables were pooled as odds ratios (ORs) using the Mantel–Haenszel method.

**Results:**

Sixty-five studies with 19 870 patients were included; 14 404 patients were classified as having low and 4975 high PD-L1 expression. High PD-L1 was associated with achieving a pathological complete response following neoadjuvant chemotherapy (OR 3.30, 95 per cent confidence interval 1.19 to 9.16; *P < *0.01; *I*^2^ = 85 per cent). Low PD-L1 expression was associated with human epidermal growth factor receptor 2 (OR 3.98, 1.81 to 8.75; *P *<* *0.001; *I*^2^ = 96 per cent) and luminal (OR 14.93, 6.46 to 34.51; *P *<* *0.001; *I*^2^ = 99 per cent) breast cancer subtypes. Those with low PD-L1 had favourable overall survival rates (HR 1.30, 1.05 to 1.61; *P *=* *0.02; *I*^2^ = 85 per cent).

**Conclusion:**

Breast cancers with high PD-L1 expression are associated with aggressive clinicopathological and immunohistochemical characteristics and are more likely to achieve a pathological complete response following neoadjuvant chemotherapy. These breast cancers are, however, associated with worse overall survival outcomes.

## Teaser

Uncertainty currently exists regarding the relevance of programmed cell death ligand 1 (PD-L1) in breast cancer. This updated systematic review and meta-analysis of 65 studies illustrates the predictive role of PD-L1 in determining pathological response rates to neoadjuvant chemotherapy, as well as its inherent value in predicting survival outcomes in breast oncology.

**Lay summary**

This is a pooled analysis assessing the relevance of a biological marker known as programmed cell death ligand 1 (PD-L1) from almost 20 000 patients diagnosed with breast cancer. The main results from this analysis suggest that high levels of PD-L1 are associated with worse survival but better response to chemotherapy for patients diagnosed with breast cancer. This may be used prospectively to enhance outcomes for patients with breast cancer.

## Introduction

Breast cancer is the most commonly diagnosed cancer and leading cause of cancer death in women worldwide. There has been an increasing incidence of breast cancer in recent decades, but breast cancer-associated mortality is decreasing^1^. This emerging trend is likely due to an increased understanding of breast cancer biology, as well as excellent progress in the development of breast cancer diagnostics and treatments^2^^,^^3^. Breast cancer treatment has evolved in the molecular area such that each subtype is managed in a manner complementary to its underlying biological drivers. Breast cancer management currently involves various combinations of surgical, chemical, radiation, hormonal and targeted therapies, although novel therapeutic avenues including immune oncology are currently being explored^4^.

Programmed cell death 1 (PD-1) plays a critical role in cancer immunotherapy; PD-1 is a receptor expressed on the surfaces of various immune cells such as T, B, and natural killer cells, which are responsible for regulation of cell death and apoptosis. Programmed cell death ligand 1 (PD-L1) (also referred to as B7-H1 or CD274) is a complementary ligand of PD-1 which is expressed on the exterior surface of cancer cells and recruited immune cells, such as macrophages and dendritic cells. PD-L1 suppresses the immunological cascade attacking the cancer cells by inducing apoptosis of local T cells, and propagating tumour proliferation as a consequence^5^^,^^6^. Manipulation of the PD-1/PD-L1 pathway by monoclonal antibodies in cancers has provided promising therapeutic approaches. Various reports^7–10^ have implied a strong correlation between PD-L1 expression and poor prognosis, particularly in malignant melanoma, and non-small cell lung, colorectal, and bladder carcinomas.

The advent of immune oncology, including immunomodulatory therapies such as immune checkpoint inhibitors (ICI), appears to hold particular promise for future clinical oncological practice, although the current role of PD-L1 assessment in breast cancer remains unclear. Traditionally, breast cancer was not considered an especially immunogenic tumour. However, recent developments have shown that some aggressive triple negative breast cancers (TNBC) are immunogenic, exhibit chemo-resistance and have a poor prognosis^11^^,^^12^. These cancers have been shown to express molecules identified as targets for immunotherapy^13^. Previous meta-analyses^14^^,^^15^ provided conflicting evidence concerning the prognostic value of PD-L1 expression for those diagnosed with invasive breast cancer, and its role as a predictive marker in neoadjuvant chemotherapy (NACT) and immunotherapy has yet to be fully elucidated. Consequently, the relevance and overall role of PD-L1 assessment in breast cancer management remains unclear.

The main aim of the present study was to perform an updated systematic review and meta-analysis evaluating the relationship between PD-L1 expression and routine clinicopathological and immunohistochemical characteristics in patients diagnosed with breast cancer, irrespective of molecular subtype. Other aims were to determine the prognostic value of PD-L1 status regarding oncological and survival outcomes, and its value as a predictor of response to current multimodal management of breast cancer.

## Methods

### Search strategy

A systematic review was undertaken in accordance with the PRISMA checklist^16^. Local institutional ethical approval was not required. An electronic search was performed of the PubMed MEDLINE, Embase, and Scopus databases on 13 June 2020 for relevant studies that would be suitable for inclusion. The search was performed for the following headings: (PD-L1 OR B7-H1 OR CD274 OR ‘programmed cell death 1 ligand 1’ OR ‘PD-L1 costimulatory protein’ OR ‘B7 homolog 1’ OR ‘B7-H1 antigen’ OR ‘CD274 antigen’) AND (“breast cancer” OR “breast neoplasms” OR “breast tumour” OR “breast carcinoma” OR “cancer of breast” OR “human mammary neoplasm” OR “human mammary carcinoma”). Only manuscripts published in the English language were included. Inclusion was not restricted based on year of publication. All titles were initially screened, and studies deemed appropriate had abstracts and full texts reviewed.

### Inclusion and exclusion criteria

Studies meeting the following criteria were included: studies on patients with histologically confirmed primary breast cancer; studies that evaluated tumour expression of PD-L1 in breast cancer tissue; and studies investigating the correlation between PD-L1 and clinicopathological parameters, treatment characteristics, pathological complete response (pCR) rates in breast tissue following NACT, disease-free survival (DFS) or overall survival (OS). Exclusion criteria were: studies only describing PD-L1 levels on tumour-infiltrating lymphocytes (TILs); studies in which patients were treated with PD-1/PD-L1-targeting therapies; review articles; studies including fewer than five patients or case reports; editorial articles; and conference abstracts.

### Data extraction and quality assessment

The literature search was undertaken by two independent reviewers using the predesigned search strategy. Duplicate studies were removed manually. Each reviewer read the retrieved manuscripts to ensure that all inclusion criteria were met, before extracting the following data: first author name, year of publication, study design, country, level of evidence, study title, number of patients, patient PD-L1 status, clinicopathological characteristics, treatment characteristics, and survival data. Data specific to patient outcomes and survival (expressed as hazard ratios (HRs), with 95 per cent c.i. and *P* values) were extracted directly from tables and study text. Risk-of-bias and methodological quality assessment was performed in accordance with the Newcastle–Ottawa scale^17^. In the event of discrepancies in opinion between the reviewers, a third reviewer was asked to arbitrate.

### Statistical analysis

PD-L1 expression and clinicopathological and treatment characteristics were presented as proportions using descriptive statistics. DFS and OS were expressed as HRs, and were considered the primary analytical endpoints. HRs and corresponding confidence intervals were retrieved directly for use in this meta-analysis. Either fixed- or random-effects models were applied on the basis of whether significant heterogeneity (*I*^2^ over 50 per cent) existed between studies included in each analysis. Symmetry of funnel plots was used to assess publication bias. Statistical heterogeneity was determined using *I*^2^ statistics. *P *<* *0.050 was considered statistically significant. Statistical analysis was done using Review Manager (RevMan) version 5.4 (Nordic Cochrane Centre, Copenhagen, Denmark).

## Results

### Literature search

**Fig. 1:**
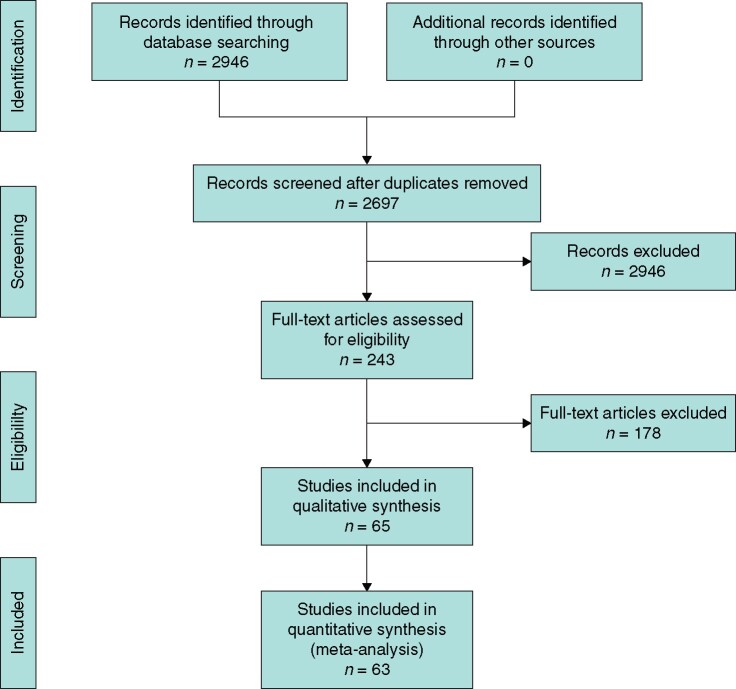
PRISMA diagram showing selection of studies for review

The initial electronic search resulted in a total of 2946 studies. After removal of 249 duplicates, the remaining 2697 titles were screened for relevance, and the abstracts and full text of 243 of these were assessed for eligibility. Overall, 65 clinical studies were included in the systematic review^18–82^, and 63 in the meta-analyses (*Fig. 1*). Individual studies included in the analysis are outlined in *Table S1*.

### Study characteristics

There were 19 870 patients included; of these, 14 404 patients had low PD-L1 expression on breast cancer cells, and 4975 had high PD-L1 expression. The median age at diagnosis was 52 years and studies had a median follow-up of 75.0 months. Molecular subtype was reported for 17 684 patients (89.0 per cent); 8532 had oestrogen receptor (ER)-positive disease (luminal type) (48.3 per cent), 6548 had TNBC (37.0 per cent), and 2604 human epidermal growth factor receptor 2 (HER2)-positive (HER2+) breast cancer (14.7 per cent).

### Clinicopathological and immunohistochemical data

Increased tumour grade (*P *<* *0.001), increased tumour stage (*P *<* *0.001), ER negativity (*P *<* *0.001), progesterone receptor (PgR) negativity (*P *<* *0.001), HER2 positivity (*P *=* *0.001), and Ki-67 proliferation index exceeding 14 per cent (*P *<* *0.001) were all independently associated with PD-L1 status (all Fisher’s exact test) (*Tables S2–S4*).

High PD-L1 expression on breast tumour cells was associated with grade 3 tumours (odds ratio (OR) 2.16, 95 per cent c.i. 1.64 to 2.83; *P *<* *0.01; *I*^2^ = 82 per cent), ER negativity (OR 2.29, 1.54 to 3.41; *P *<* *0.001; *I*^2^ = 89 per cent), PgR negativity (OR 2.44, 1.69 to 3.51; *P *<* *0.001; *I*^2^ = 83 per cent), and Ki-67 index greater than 14 per cent (OR 2.12, 1.23 to 3.65; *P *=* *0.007; *I*^2^ = 89 per cent) ( *Fig. S1*). *Fig. S2* provides details for all clinicopathological and immunohistochemical variables that failed to reach statistical significance in meta-analyses.

### Breast cancer molecular subtypes

**Fig. 2:**
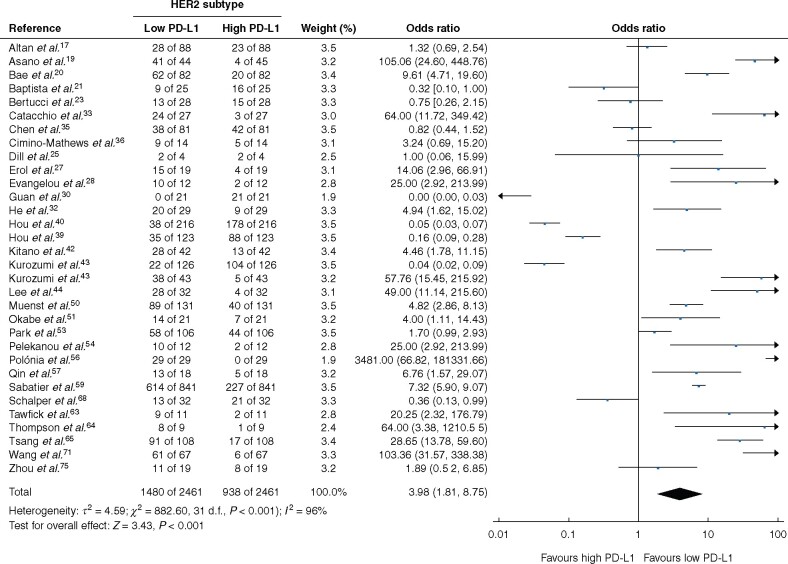
Association between programmed cell death ligand 1 expression and human epidermal growth factor receptor 2-amplified molecular subtype of breast cancer A Mantel–Haenszel random-effects model was used for meta-analysis. Odds ratios are shown with 95 per cent confidence intervals. PD-L1, programmed cell death ligand 1. Kurozumi et al. evaluated two independent patient cohorts hence being twice in this analysis.

**Fig. 3:**
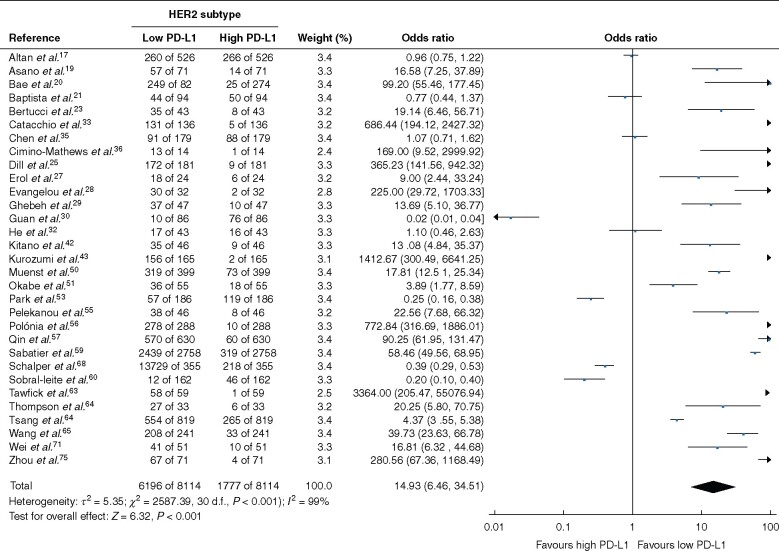
Association between programmed cell death ligand 1 expression and luminal molecular subtype type of breast cancer A Mantel–Haenszel random-effects model was used for meta-analysis. Odds ratios are shown with 95 per cent confidence intervals. PD-L1, programmed cell death ligand 1.

Low PD-L1 expression was associated with the HER2 (OR 3.98, 95 per cent c.i. 1.81 to 8.75; *P *<* *0.001; *I*^2^ = 96 per cent) and luminal (OR 14.93, 6.46 to 34.51; *P *<* *0.001; *I*^2^ = 99 per cent) molecular subtypes (*Figs 2* and *3*). PD-L1 expression was not associated with TNBC (OR 0.98, 0.74 to 1.30; *P *=* *0.90; *I*^2^ = 97 per cent), and failed to inform patient prognosis in terms of DFS or OS for patients with TNBC (*Fig. S2d,g,h*). Performing subgroup survival analyses for the luminal and HER2 molecular subgroups was not feasible owing to lack of available data.

### Treatment characteristics

**Fig. 4:**
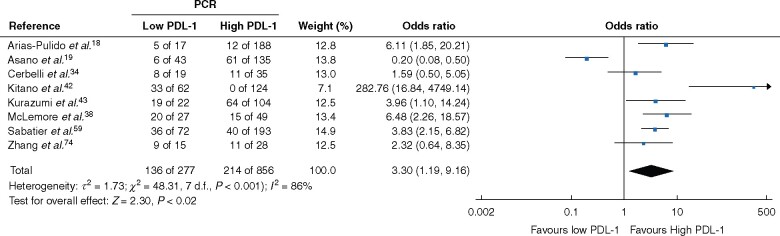
Association between programmed death ligand 1 expression and likelihood of achieving a pathological complete response after neoadjuvant chemotherapy A Mantel–Haenzel random-effects model was used for meta-analysis. Odds ratios are shown with 95 per cent confidence intervals. pCR, pathological complete response; PD-L1, programmed cell death ligand 1.

Thirteen studies reported on breast cancer management using treatment with NACT and PD-L1 expression. Eleven studies reported patients achieving a pCR in the breast following NACT, eight of which were suitable for meta-analysis. Patients with high PD-L1 expression were more likely to achieve a pCR after NACT (OR 3.30, 1.19 to 9.16; *P < *0.01; *I*^2^ = 85 per cent) (*Fig. 4*).

Three studies reported adjuvant chemotherapy prescription with respect to PD-L1. Adjuvant chemotherapy was prescribed for 759 of 952 patients (79.7%) with low PD-L1 expression and 333 of 543 (61.3%) with high PD-L1 expression (*P *<* *0.001). Details regarding adjuvant radiotherapy and PD-L1 expression was recorded in three studies; 90 of 952 patients with low PD-L1 expression and 88 of 543 with high PD-L1 expression received radiotherapy (*P *<* *0.001). Only AiErken and colleagues^19^ reported results regarding PD-L1 status and surgical management; all 70 patients with PD-L1-positive disease underwent mastectomy, whereas 136 of 145 (93.8 per cent) with PD-L1-negative disease underwent mastectomy (*P* = 0.027). Treatment characteristics and associations between PD-L1 and responses to NACT are outlined in *Tables S5* and *S6*.

### Survival outcomes

**Fig. 5:**
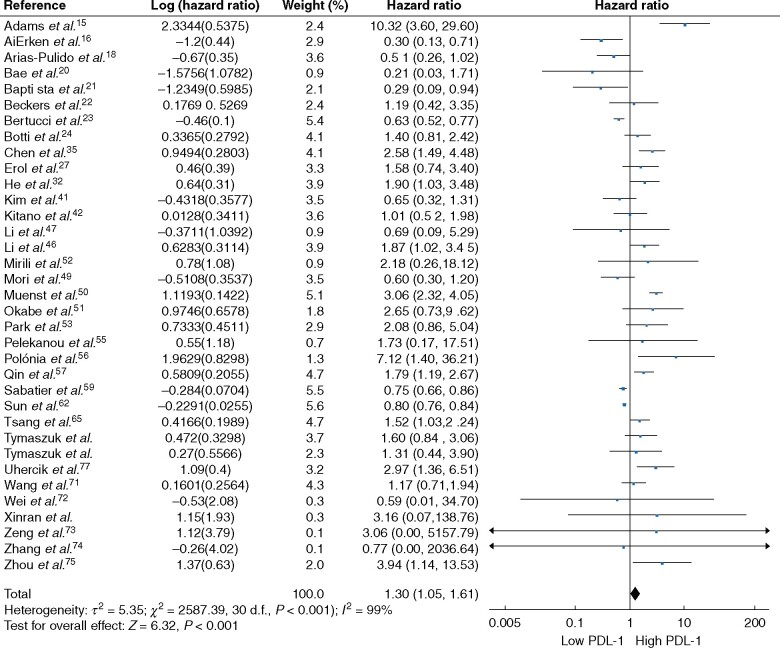
Association between programmed death ligand 1 expression and overall survival in breast cancer An inverse-variance random-effects model was used for meta-analysis. Hazard ratios are shown with 95 per cent confidence intervals.

Patients with cancers expressing high levels of PD-L1 on breast tumour cells had worse OS rates than those with low PD-L1 expression (HR 1.30, 95 per cent c.i. 1.05 to 1.61; *P *=* *0.02). There was significant heterogeneity between the 35 included independent patient cohorts (*I*^2^ = 85 per cent; *P *<* *0.001) (*Fig. 5*).

There was no increased risk of breast cancer recurrence for patients with high PD-L1 expression *versus* those with low PD-L1 expression on breast cancer cells (HR 1.11, 0.86 to 1.44; *P *=* *0.41). Significant heterogeneity was present between the 26 patient cohorts analysed for DFS (*I*^2^ = 88 per cent; *P *<* *0.001) (*Fig. S2e*). Furthermore, there was no increased risk of disease recurrence after 5 years for those with high PD-L1 expression (HR 0.86, 0.64 to 1.17; *P *=* *0.34; *I*^2^ = 73 per cent, *P *<* *0.001) (*Fig. S2f*).

## Discussion

The present analysis of 19 870 patients with breast cancer investigated the prognostic value of PD-L1 expression on breast cancer cells. The most important clinical findings are the increased likelihood of achieving a pCR in tumours expressing high levels of PD-L1 on cancer cells, as well as the favourable OS outcomes for patients with breast cancers expressing low PD-L1 levels. Breast oncology has evolved in concordance with the molecular era, such that biomarkers, genetic testing, and genomic assays are used to personalize treatment regimens and provide valuable prognostic information; immunomodulatory strategies are a promising approach to enhance outcomes even further^83^^,^^84^. This study provides clarity pertaining to the clinical role of PD-L1 immunohistochemical testing; incorporation of this biomarker into routine histopathological evaluation of diagnostic tissue biopsies may aid clinical and therapeutic decision-making in the neoadjuvant setting, and further reduce prognostic uncertainty of a breast cancer diagnosis.

Evidence from this meta-analysis suggests that increased PD-L1 expression by cancer cells indicates that patients are likely to achieve a pCR on NACT. A pCR has been recognized as an excellent indicator of prognosis and survival in breast cancer in a number of prospective studies^85^, with patients attaining a pCR typically outperforming their counterparts in terms of survival. These results support the clinical importance of clarifying PD-L1 status on core tissue biopsy in the neoadjuvant setting in those being considered for preoperative systemic chemotherapy. Furthermore, recent prospective studies such as the IMPASSION 130 and Keynote 522 trials^86^^,^^87^ have outlined the role of confirming PD-L1 status on tissue biopsy in both early-stage and metastatic settings, when gauging whether immunotherapeutic agents, such as anti-PD-L1 drugs, will be indicated. These novel immunotherapies currently rely on clarification of PD-L1 status^88^, and their potential for enhancing oncological and survival outcomes for even the most aggressive of TNBCs has been described^87^. This data, reported in tandem with these large prospective studies, support routine PD-L1 detection in breast cancer immunohistochemical tissue analysis, given the robust prognostic value PD-L1 offers within the breast oncological paradigm, as well as its potential role as a prospective therapeutic target.

In contrast, the results shown here from a pooled analysis of 33 independent patient cohorts highlight a significant survival benefit associated with breast cancer diagnoses harbouring low PD-L1 expression on cancer cells. These observations are consistent with two previous meta-analyses^14^^,^^89^, both of which suggested that high PD-L1 expression is correlated with poor prognosis. These results imply that PD-L1 could perhaps be considered as a reliable predictor of OS in breast cancer, even though this is incongruent with observations associating PD-L1 expression to pCR, a recognised surrogate for survival^85^. While this inconsistency creates ambiguity over the interpretation of PD-L1 expression and its supplementary value in breast oncology, perhaps increased PD-L1 expression provides a novel avenue of investigation to establish the rationale surrounding the cohort of patients who paradoxically successfully achieve a pCR, yet fail to derive meaningful long-term survival benefit from therapy, as alluded to in several prospective analyses^85^.

Although PD-L1 provides excellent prognostic information regarding pCR and patient mortality, its relevance as an indicator of disease recurrence is less conclusive. Both previous meta-analyses reported PD-L1 as a predictive biomarker of DFS, a finding that was not replicated here, and uncertainty is further potentiated as PD-L1 failed to predict DFS within TNBC. Huang and colleagues described PD-L1 positivity correlating with earlier time to breast cancer recurrence in patients managed with breast-conserving surgery followed by radiotherapy^90^, further complicating the application of PD-L1 in breast oncology. There is a temptation to implicate the aggressive clinicopathological characteristics associated with increased PD-L1 expression on cancer cells as providing the rationale for worse OS. In the absence of disease recurrence, however, the present data fail to support this. Data surrounding a systemic role of PD-L1 suggest that host immunological factors and other complex factors may contribute, as upregulation of PD-L1 suppresses T lymphocyte function overall, increasing patient susceptibility to infection and inflammation^91^. Moreover, simple measures of PD-L1 expression does not capture differential enrichments across patients, tumour and immune cell subtypes, as well as the spatial proximity of these cell types in tissues. These relational features may be critical to further evaluating the complex stimulatory and inhibitory processes that depends on the interplay between individual cells in the tumour microenvironment, and the immune system.^92^ In the interim, prognostic information provided by PD-L1 assessment concerning pCR and mortality is explicit, and supports its potential value as a breast cancer biomarker.

In this meta-analysis, increased PD-L1 expression correlated with aggressive clinicopathological and immunohistochemical tumour features, such as grade 3 tumours, ER and PgR negativity, as well as Ki-67 proliferation greater than 14 per cent. Uncontrolled proliferation is a hallmark of oncogenesis^93^, and consequently, routine Ki-67 proliferation staining is performed to appraise the proportion of tumour cells actively proliferating at a molecular level^94^. Ki-67 indices, as well as the mitotic component of Nottingham histopathological grading systems, are of crucial clinical relevance in modern histopathological reporting^95^. Moreover, ER and PgR negativity both significantly correlated with PD-L1 expression in this analysis, indicating that PD-L1 status provides data somewhat compatible with routine prognostic molecular variables. This complements the findings concerning OS, particularly when historical data connect ER and PgR negativity, increased histological grade, and Ki-67 proliferation to worse clinical outcomes in breast cancer^96–98^. Linking increased PD-L1 expression with aggressive microscopic tumour characteristics and immunohistochemical features encourages hypotheses that assessment of PD-L1 would be best reserved for those with triple-negative and ER-positive/PgR-negative disease, whose responses to systemic treatments fall short of their ER-positive/PgR-positive and HER2-positive counterparts. Perhaps ICIs have the potential to bridge the current gap in clinical outcomes between these molecular subtypes, and the authors await the results of the IMPASSION 130 and Keynote 522 trials with great anticipation^86^^,^^87^.

The results of the present study lead to a congruent message: evaluating PD-L1 status appears advantageous in reducing prognostic uncertainty for patients diagnosed with breast cancer. Reduced PD-L1 in the luminal and HER2+ molecular subtypes corresponds with their more favourable prognoses^64^; however, the challenge remains in establishing the scientific reasoning for such results. Within the tumour microenvironment, interplay between PD-1 expressed on TILs and its complementary ligand (expressed on both cancer and immune cells) appears to suppress and dampen the cytotoxic anticancer effect of local inflammatory cells^99^. Consequently, cancer development may be unopposed by the host immune system, even in more immunogenic breast cancer subtypes such as TNBC, leading to development of more aggressive tumour biology^100^. This provides a rationale for the strong associations between high PD-L1 expression and aggressive clinicopathological characteristics, and moreover, the worse survival outcomes in the long term. On the contrary, the paradigm is evolving such that positive PD-L1 expression may provide the potential for novel therapeutic avenues for patients in both early and metastatic settings^86^^,^^87^, which perhaps suggests PD-L1 positivity may develop into a favourable parameter in certain circumstances. In the interim,the results of this meta-analysis demonstrate high PD-L1 expression expression is an adverse prognostic marker in its current unmanipulated state, as well as having indispensable value in predicting those likely to achieve a pCR during NACT.

Despite efforts to ascertain accurate and comprehensive information with clinical relevance, several limitations should be considered when interpreting the results of this analysis. The study was based solely on studies published in the English language, and the vast majority of included studies were conducted retrospectively, providing low-to-moderate levels of evidence. The included studies provided inconsistent definitions and cut-offs defining PD-L1 status, and there was considerable variation in determining PD-L1 expression, such as in detection antibodies used and preparation of tissues, and human error in reporting. Perhaps expert consensus, such as those proposed annually by the St Gallen panel or the recent standardised methodology to assess TILs in breast cancer^101^, may shed light on the most appropriate means of evaluating PD-L1 expression. Survival analysis was not available for 58 per cent of included studies (38 of 65), limiting the conclusions that can be drawn from these analyses. The paucity of studies reporting on HER2+ or luminal molecular subtypes in isolation limited independent molecular subtype analyses of PD-L1 status and survival outcomes. This review focused solely on PD-L1 expression by cancer cells, and provides no prognostic information regarding the role of PD-L1 status on tumour microenvironmental immune cells; debate about which is more critical in predicting clinical outcomes and response to ICI is ongoing. Moreover, as previously mentioned simple measures of PD-L1 expression does not capture the complexity of the relational features within the tumour microenvironment that may be critical to further evaluating the complex and dynamic dual tumour stimulatory and inhibitory processes of the immune system on breast cancer tumourogenesis and establishing the basis for novel individualised cancer immunotherapies, including ICIs. 

*Disclosure.* The authors have no conflicts of interest to declare.

## Supplementary material

Supplementary material is available at *BJS* online.

## Supplementary Material

znab103_Supplementary_Data
