# Supplementary material for: Clinicopathological and prognostic significance of programmed cell death ligand 1 expression in patients diagnosed with breast cancer: meta-analysis
Source: Br J Surg. 2021 May 8;108(6):622–31. doi: 10.1093/bjs/znab103 (PMC10364926; doi:10.1093/bjs/znab103)
Supplement: znab103_Supplementary_Data [file znab103_supplementary_data.zip › Table S6.docx]

|  | *Low PDL1* | *High PDL1* | *p-value* |
| --- | --- | --- | --- |
| *NACT*  *pCR*  *PR* | *1150*  *246*  *189* | *461*  *201*  *168* | *<0.001**  *<0.001** |

*NAC; neoadjuvant chemotherapy, pCR; pathological complete response, PR; partial pathological response, PD-L1; programmed death ligand-1*

**Table S6** Associations of neoadjuvant chemotherapy responses and programmed death ligand-1 expression on tumour cells
