# Supplementary material for: Clinicopathological and prognostic significance of programmed cell death ligand 1 expression in patients diagnosed with breast cancer: meta-analysis
Source: Br J Surg. 2021 May 8;108(6):622–31. doi: 10.1093/bjs/znab103 (PMC10364926; doi:10.1093/bjs/znab103)
Supplement: znab103_Supplementary_Data [file znab103_supplementary_data.zip › Table S5.docx]

| Author | Year | NACT (L) | NACT (H) | pCR (L) | pCR (H) | PR (L) | PR (H) | AC (L) | AC (H) | XRT (L) | XRT (H) |
| --- | --- | --- | --- | --- | --- | --- | --- | --- | --- | --- | --- |
| AiErken | 2017 | 14 | 4 | . | . | . | . | 133 | 65 | 35 | 13 |
| Arias-Pulido | 2018 | 188 | 17 | 12 | 5 | . | . | . | . | . | . |
| Asano | 2018 | 135 | 42 | 61 | 6 | . | . | . | . | . | . |
| Cerbelli | 2017 | 35 | 19 | 11 | 8 | . | . | . | . | . | . |
| Chen | 2017 | 156 | 153 | 0 | 0 | 156 | 153 |  |  |  |  |
| Hou (3) | 2017 | 58 | 6 | 7 | 32 | 14 | 11 | . | . | . | . |
| Kitano | 2017 | 124 | 62 | 0 | 33 | . | . | . | . | . | . |
| Kurazumi (B) | 2019 | 104 | 22 | 64 | 19 | . | . | . | . | . | . |
| McLemore | 2018 | 49 | 27 | 15 | 20 | . | . | . | . | . | . |
| Pelekanou (2) | 2018 | . | . | 25 | 33 | . | . | . | . | . | . |
| Qin | 2015 | 56 | 19 | . | . | . | . | 603 | 175 | 23 | 8 |
| Sabatier | 2015 | 193 | 72 | 40 | 36 | . | . | . | . | . | . |
| Sobral-Leite | 2018 | . | . | . | . | . | . | 23 | 93 | 32 | 67 |
| Zhang | 2019 | 28 | 15 | 11 | 9 | 19 | 4 | . | . | . | . |
| Zhu | 2018 | 10 | 3 | . | . | . | . | . | . | . | . |
| Total |  | 1150 | 461 | 246 | 201 | 189 | 168 | 759 | 333 | 90 | 88 |

*NACT; neoadjuvant chemotherapy, pCR; pathological complete response, PR; partial pathological response, AC; adjuvant chemotherapy, XRT; adjuvant radiotherapy, L; low programme death ligand-1 expression, H; high programme death ligand-1 expression.*

**Table S5** Table illustrating the frequency of high and low programme death ligand-1 expression for the various treatment characteristics for patients from 15 independent patient cohorts of the 65 studies included in this systematic review.
