# Supplementary material for: Clinicopathological and prognostic significance of programmed cell death ligand 1 expression in patients diagnosed with breast cancer: meta-analysis
Source: Br J Surg. 2021 May 8;108(6):622–31. doi: 10.1093/bjs/znab103 (PMC10364926; doi:10.1093/bjs/znab103)
Supplement: znab103_Supplementary_Data [file znab103_supplementary_data.zip › Table S4.docx]

|  | *Low PDL1* | *High PDL1* | *p-value* |
| --- | --- | --- | --- |
| ***Histological subtype***  *IDC* | *4027* | *1149* |  |
| *Other subtypes* | *781* | *219* | *0.835* † |
| ***Grade***  *Grade 1 / 2*  *Grade 3* | *5261*  *4375* | *1335*  *2061* | *<0.001** † |
| ***Tumour stage***  *T1 / 2*  *T 3 / 4* | *4166*  *925* | *1309*  *375* | *<0.001** † |
| ***Lymph node metastasis***  *Positive*  *Negative* | *4491*  *4643* | *1677*  *1741* | *0.917* † |
| ***Estrogen expression*** |  |  |  |
| *Positive*  *Negative* | *7236*  *2204* | *1633*  *1265* | *<0.001** † |
|  |  |  |  |
| ***Progesterone expression*** |  |  |  |
| *Positive*  *Negative* | *4395*  *3186* | *856*  *1307* | *<0.001** † |
|  |  |  |  |
| ***HER2 expression*** |  |  |  |
| *HER2+*  *HER2-* | *1381*  *6551* | *515*  *1924* | *<0.001** † |
| ***Ki-67 proliferation index***  *Ki67 >14%*  *Ki67 <14%* | *3066*  *3556* | *1197*  *599* | *<0.001** † |

*IDC; invasive ductal carcinoma, HER2; human epidermal growth factor-2, T; tumour stage*

†; Fisher’s exact test

**Table S4** Details relating to the associations between routine clinicopathological and immunohistochemical characteristics and programmed death cell ligand-1 expression on breast cancer tumour cells.
