# Supplementary material for: Clinicopathological and prognostic significance of programmed cell death ligand 1 expression in patients diagnosed with breast cancer: meta-analysis
Source: Br J Surg. 2021 May 8;108(6):622–31. doi: 10.1093/bjs/znab103 (PMC10364926; doi:10.1093/bjs/znab103)
Supplement: znab103_Supplementary_Data [file znab103_supplementary_data.zip › Table S3.docx]

| Author | Year | ER+ (L) | ER+ (H) | PR+ (L) | PR+ (H) | HER2+ (L) | HER2+ (H) | Ki67>14 (L) | Ki67>14 (H) |
| --- | --- | --- | --- | --- | --- | --- | --- | --- | --- |
| Altan | 2018 | . | 58 | . | . | . | . | . | . |
| Asano | 2018 | . | . | . | . | . | . | 77 | 26 |
| Bae | 2016 | 286 | 25 | 154 | 11 | 62 | 20 | 152 | 57 |
| Baptista | 2015 | 50 | 44 | 41 | 48 | 9 | 16 | . | . |
| Bertucci | 2015 | 46 | 18 | 38 | 19 | 22 | 11 | . | . |
| Catacchio | 2019 | 131 | 5 | 103 | 3 | 24 | 3 | . | . |
| Chen | 2017 | 91 | 88 | 68 | 75 | 39 | 42 | . | . |
| Cimino-Matthews | 2016 | 13 | 1 | . | . | 9 | 5 | . | . |
| Dill | 2018 | 153 | 8 | 153 | 8 | 2 | 2 | . | . |
| Evangelou | 2020 | 30 | 2 | 34 | 4 | 10 | 2 | . | . |
| Ghebah | 2007 | 37 | 10 | 28 | 6 | . | . | . | . |
| Guan | 2016 | 10 | 73 | 9 | 70 | 0 | 20 | . | . |
| He | 2018 | 15 | 9 | 14 | 7 | 20 | 9 | . | . |
| Hou | 2019 | . | . | . | . | 186 | 11 | . | . |
| Hou | 2018 | 114 | 12 | . | . | . | . | . | . |
| Hou | 2017 | 77 | 11 | 78 | 10 | . | . | . | . |
| Kitano | 2017 | 35 | 9 | . | . | . | . | . | . |
| Kurazumi A | 2019 | 165 | 2 | 141 | 3 | 38 | 5 | . | . |
| Kurazumi B | 2019 | 42 | 7 | 30 | 5 | . | . | . | . |
| Lee | 2019 | 305 | 2 | . | . | 28 | 4 | . | . |
| Muenst | 2013 | 365 | 92 | . | . | 89 | 40 | 389 | 136 |
| Okabe | 2017 | 36 | 18 | . | . | 14 | 7 | . | . |
| Pelekanou (1) | 2017 | 38 | 8 | . | . | 10 | 2 | . | . |
| Polonia | 2017 | 253 | 8 | 184 | 7 | 55 | 4 | 21 | 4 |
| Qin | 2015 | 570 | 60 | 555 | 62 | 13 | 5 | 349 | 91 |
| Sabatier | 2015 | 3290 | 512 | 2406 | 392 | 525 | 170 | 1789 | 665 |
| Schalper | 2014 | 137 | 218 | . | . | 13 | 19 | . | . |
| Sobral-Leite | 2018 | 28 | 15 | 79 | 91 | 27 | 74 | . | . |
| Sun | 2016 | . | . | . | . | . | . | 100 | 70 |
| Tawfik | 2018 | 58 | 1 | 40 | 1 | 9 | 2 | . | . |
| Tsang | 2017 | 554 | 265 | . | . | 91 | 17 | . | . |
| Wei | 2020 | 41 | 10 | . | . | . | . | . | . |
| Zeng | 2019 | . | . | . | . | . | . | 62 | 44 |
| Zhou | 2018 | 54 | 14 | 48 | 11 | 27 | 19 | 29 | 4 |
| Total |  | 7246 | 1633 | 4395 | 856 | 1383 | 515 | 3066 | 1197 |

*ER+; oestrogen receptor positive breast cancer, PR+; progesterone receptor positive breast cancer, HER2; human epidermal growth factor receptor-2 positive breast cancer, L; low programme death ligand-1 expression, H; high programme death ligand-1 expression.*

**Table S3** Table illustrating the frequency of high and low programme death ligand-1 expression for immunohistochemical molecular markers of breast cancer in 33 independent patient cohorts of the 65 studies included in this systematic review.
