# Supplementary material for: Clinicopathological and prognostic significance of programmed cell death ligand 1 expression in patients diagnosed with breast cancer: meta-analysis
Source: Br J Surg. 2021 May 8;108(6):622–31. doi: 10.1093/bjs/znab103 (PMC10364926; doi:10.1093/bjs/znab103)
Supplement: znab103_Supplementary_Data [file znab103_supplementary_data.zip › Table S2.docx]

| Author | Year | IDC (L) | IDC (H) | Grade 3 (L) | Grade 3 (H) | T3/4 (L) | T3/4 (H) | Node+ (L) | Node+ (H) |
| --- | --- | --- | --- | --- | --- | --- | --- | --- | --- |
| AiErken | 2017 | . | . | . | . | 27 | 15 | 65 | 27 |
| Altan | 2018 | . | . | . | . | 75 | 6 | . | . |
| Asano | 2018 | . | . | 29 | 11 | . | . | 104 | 32 |
| Bae | 2016 | 364 | 59 | 127 | 47 | 24 | 3 | 157 | 14 |
| Baptista | 2015 | . | . | 49 | 87 | . | . | 40 | 87 |
| Bertucci | 2015 | 62 | 39 | 47 | 32 | . | . | . | . |
| Botti | 2017 | 120 | 71 | 116 | 69 | 13 | 5 | 58 | 18 |
| Catacchio | 2019 | 135 | 5 | 52 | 4 | . | . | 63 | 2 |
| Cerbelli | 2017 | . | . | 35 | 19 | . | . | . | . |
| Chen | 2017 | . | . | 28 | 24 | 107 | 92 | 118 | 110 |
| Cimino-Matthews | 2016 | . | . | 22 | 9 | . | . | . | . |
| Dill | 2018 | 157 | 22 | 57 | 25 | 19 | 1 | 18 | 2 |
| Dogukan | 2019 | 32 | 10 | 31 | 19 | 7 | 8 | 20 | 11 |
| Erol | 2019 | . | . | . | . | . | . | 3 | 5 |
| Evangelou | 2020 | . | . | 19 | 9 | 3 | 1 | 17 | 4 |
| Ghebah | 2007 | . | . | 19 | 14 | . | . | . | . |
| Guan | 2016 | . | . | 4 | 25 | 2 | 40 | 5 | 47 |
| Guo | 2016 | 146 | 21 | 85 | 24 | . | . | 54 | 8 |
| He | 2018 | 37 | 24 | 34 | 20 | . | . | 37 | 22 |
| Hou 2 | 2018 | 163 | 37 | 100 | 35 | 17 | 0 | 90 | 12 |
| Hou 3 | 2017 | 67 | 21 | 13 | 55 | . | . | 7 | 25 |
| Kim | 2020 | . | . | 47 | 15 | 57 | 14 | 34 | 12 |
| Kitano | 2017 | . | . | 64 | 47 | 40 | 39 | 35 | 45 |
| Kurazumi (1) | 2019 | . | . | 125 | 20 | 110 | 11 | 101 | 10 |
| Kurazumi (2) | 2019 | . | . | 85 | 22 | 38 | 6 | 68 | 17 |
| Li (1) | 2018 | 90 | 22 | . | . | 28 | 13 | 42 | 16 |
| Li (2) | 2018 | . | . | . | 22 | . | . | . | 26 |
| Lou | 2017 | 40 | 24 | 25 | 21 |  |  | 19 | 11 |
| Mori | 2017 | . | . | 88 | 80 | 5 | 3 | 44 | 36 |
| Muenst | 2013 | . | . | 170 | 78 | 74 | 45 | 208 | 86 |
| Okabe | 2017 | . | . | 13 | 9 | . | . | 30 | 15 |
| Pelekanou (1) | 2017 | 42 | 8 | 15 | 6 | . | . | 29 | 8 |
| Polonia | 2017 | . | . | 179 | 20 | 48 | 4 | 168 | 10 |
| Qin | 2015 | . | . | 370 | 125 | 91 | 27 | 347 | 94 |
| Ren | 2018 | 165 | 12 | 123 | 11 | 8 | 0 | 80 | 2 |
| Sabatier | 2015 | 2315 | 554 | 1487 | 531 | . | . | 1559 | 398 |
| Schalper | 2014 | . | . | 36 | 78 | . | . | 50 | 81 |
| Sobral-Leite | 2018 | 92 | 220 | 36 | 174 | . | . | 44 | 71 |
| Sun | 2016 | . | . | . | . | . | . | 59 | 1 |
| Tawfik | 2018 | . | . | 30 | 17 | . | . | . | . |
| Tsang | 2017 | . | . | 400 | 96 | 56 | 21 | 389 | 147 |
| Uhercik | 2017 | . | . | . | 46 | . | . | . | . |
| Wei | 2020 | . | . | 18 | 7 | . | . | 19 | 9 |
| Zeng | 2019 | . | . | 42 | 38 | . | . | 5 | 34 |
| Zhang | 2019 | . | . | . | . | 15 | 6 | 11 | 8 |
| Zhou | 2018 | . | . | 50 | 28 | 5 | 5 | 47 | 16 |
| Zhu | 2018 | . | . | 32 | 12 | . | . | . | . |
| Total |  | 4027 | 1149 | 4375 | 2061 | 925 | 375 | 4491 | 1677 |

*L; low programme death ligand-1 expression, H; high programme death ligand-1 expression,*

*IDC; invasive ductal carcinoma, Node+; nodal metastatic involvement, T3; tumour stage 3 or greater*

**Table S2** Table illustrating the frequency of high and low programme death ligand-1 expression for histopathological tumour characteristics for patients in the 47 independent patient cohorts from the 65 studies included in this systematic review.
