# Supplementary material for: Clinicopathological and prognostic significance of programmed cell death ligand 1 expression in patients diagnosed with breast cancer: meta-analysis
Source: Br J Surg. 2021 May 8;108(6):622–31. doi: 10.1093/bjs/znab103 (PMC10364926; doi:10.1093/bjs/znab103)
Supplement: znab103_Supplementary_Data [file znab103_supplementary_data.zip › Table S1.docx]

| Author | | Year | Country | Number (N) | PDL1-low | PDL1-high | PDL-1  level | PDL-1 assessment | Median age (years) | Follow up (months) | NOS |
| --- | --- | --- | --- | --- | --- | --- | --- | --- | --- | --- | --- |
| Adams | 2018 | | US | 128 | 66 | 62 | N/A | N/A | 55 | N/R | 6 |
| AiErken | 2017 | | China | 215 | 145 | 70 | Protein | IHC | 49 | 68 | 7 |
| Altan | 2018 | | US | 1034 | 915 | 120 | RNA | Microarray | N/R | N/R | 6 |
| Arias-Pulido | 2018 | | US | 221 | 154 | 18 | Protein | IHC | N/R | N/R | 6 |
| Asano | 2018 | | Japan | 177 | 135 | 42 | Protein | IHC N/R | | 41 | 8 |
| Bae | 2016 | | Korea | 465 | 402 | 63 | Protein | IHC | 52 | 41 | 8 |
| Baptista | 2015 | | Brazil | 189 | 82 | 107 | Protein | IHC | 51 | 86 | 7 |
| Beckers | 2016 | | Australia | 161 | 38 | 123 | Protein | IHC | 57 | 55 | 7 |
| Bertucci | 2015 | | France | 110 | 70 | 40 | RNA | Microarray | N/R | 43 | 7 |
| Botti | 2017 | | Italy | 238 | 161 | 77 | Protein | IHC | 57 | N/R | 6 |
| Catacchio | 2019 | | Italy | 167 | 160 | 7 | Protein | IHC | 57 | 63 | 6 |
| Cerbelli | 2017 | | Italy | 54 | 35 | 19 | Protein | IHC | 50 | N/R | 6 |
| Chen | 2017 | | China | 309 | 156 | 153 | Protein | IHC | 49 | 70 | 8 |
| Cimino-Matthews | 2016 | | US | 43 | 34 | 9 | Protein | IHC | 54 | N/R | 6 |
| Dill | 2018 | | US | 242 | 215 | 30 | Protein | IHC | N/R | N/R | 5 |
| Dogukan | 2019 | | Turkey | 61 | 38 | 23 | Protein | IHC | 50 | 25 | 5 |
| Erol | 2019 | | Turkey | 57 | 40 | 12 | Protein | IHC | 53 | 33 | 6 |
| Evangelou | 2020 | | Greece | 45 | 36 | 9 | Protein | IHC | 35 | N/R | 7 |
| Ghebah | 2007 | | Saudi Arabia | 69 | 33 | 34 | Protein | IHC | 44 | N/R | 6 |
| Guan | 2016 | | China | 134 | 15 | 119 | Protein | IHC | N/R | N/R | 5 |
| Guo | 2016 | | China | 183 | 158 | 25 | Protein | IHC N/R | | 76 | 8 |
| He | 2018 | | US | 68 | 43 | 25 | Protein | IHC | 48 | 20 | 8 |
| Hirakata | 2020 | | Japan | 97 | 80 | 17 | N/R | IHC | N/R | N/R | 5 |
| Hou | 2019 | | US | 298 | 276 | 22 | Protein | IHC | 53 | N/R | 6 |
| Hou | 2018 | | US | 216 | 178 | 38 | Protein | IHC | 53 | 73 | 9 |
| Hou | 2017 | | US | 123 | 102 | 21 | Protein | IHC | 56 | N/R | 6 |
| Kim | 2020 | | Korea | 83 | 64 | 19 | Protein | IHC | 47 | N/R | 8 |
| Kitano | 2017 | | Japan | 180 | 124 | 62 | Protein | IHC | 54 | 115 | 8 |
| Kurazumi (1) | 2019 | | Japan | 248 | 228 | 20 | N/R | IHC | N/R | 128 | 8 |
| Kurazumi (2) | 2019 | | Japan | 126 | 104 | 22 | N/R | IHC | N/R | N/R | 7 |
| Lee (1) | 2019 | | Korea | 392 | 378 | 15 | Protein | IHC | 47 | 89 | 8 |
| Lee (2) | 2019 | | Korea | 358 | 323 | 35 | Protein | IHC | 51 | N/R | 7 |
| Li (1) | 2018 | | China | 112 | 90 | 22 | Protein | IHC | N/R | N/R | 8 |
| Li (2) | 2018 | | China | 101 | 75 | 26 | Protein | IHC | 51 | 49 | 8 |
| Li | 2016 | | US | 136 | 108 | 28 | Protein | IHC N/R | | N/R | 8 |
| McLemore | 2018 | | US | 76 | N/R | N/R | Protein | IHC | 51 | N/R | 7 |
| Lou | 2017 | | China | 64 | 40 | 24 | Protein | IHC | N/R | N/R | 6 |
| Mirili | 2019 | | Turkey | 97 | 80 | 17 | Protein | IHC | 49 | 93 | 8 |
| Mori | 2017 | | japan | 248 | 145 | 103 | Protein | IHC | 60 | 68 | 7 |
| Muenst | 2013 | | Switzerland | 650 | 498 | 152 | Protein | IHC | 63 | 65 | 6 |
| Okabe | 2017 | | japan | 97 | 65 | 32 | Protein | IHC | 58 | 127 | 8 |
| Park | 2016 | | Korea | 316 | 153 | 163 | Protein | IHC | 47 | 118 | 8 |
| Pelekanou | 2018 | | US | 120 | 68 | 52 | Protein | IHC | N/R | 36 | 6 |
| Pelekanou(2) | 2017 | | US | 58 | 48 | 10 | Protein | QIF | N/R | N/R | 6 |
| Polonia | 2017 | | Portugal | 435 | 407 | 28 | Protein | IHC | 60 | 120 | 8 |
| Qin | 2015 | | China | 870 | 681 | 189 | Protein | IHC | 47 | 98 | 8 |
| Ren | 2018 | | China | 195 | 134 | 61 | RNA | RNAscope | 51 | N/R | 8 |
| Sabatier | 2015 | | France | 5454 | 4378 | 1076 | RNA | Microarrays | N/R | N/R | 6 |
| Schalper | 2014 | | US | 696 | 407 | 289 | RNA | RNAscope | N/R | 139 | 5 |
| Sobral-Leite | 2018 | | Netherlands | 410 | 126 | 284 | N/R | Microarrays | N/R | N/R | 7 |
| Sun | 2016 | | South Korea | 218 | 189 | 29 | Protein | IHC | N/R | N/R | 6 |
| Tawfik | 2018 | | US | 133 | 116 | 17 | Protein | IHC | N/R | N/R | 5 |
| Thompson | 2017 | | US | 47 | 39 | 8 | Protein | Microarray | 59 | 52 | 5 |
| Tsang | 2017 | | Hong Kong | 1091 | 796 | 295 | Protein | IHC | 55 | 63 | 7 |
| Tung | 2015 | | US | 197 | 146 | 51 | Protein | IHC | 48 | N/R | 6 |
| Tymoszuk | 2014 | | Austria/France | 132 | N/R | N/R | N/R | RT-PCR | N/R | 104 | 5 |
| Uhercik | 2017 | | UK | 127 | 37 | 90 | GS | qPCR | N/R | 120 | 8 |
| Van Berckelaer | 2019 | | Belgium | 105 | 103 | 2 | Protein | IHC | 60 | 42 | 8 |
| Xinran | 2019 | | China | 223 | 204 | 19 | Protein | IHC | 53 | 83 | 8 |
| Wang | 2017 | | Canada | 443 | 70 | 370 | Protein | IHC | N/R | 87 | 7 |
| Wei | 2020 | | China | 77 | 58 | 19 | Protein | IHC | 49 | N/R | 7 |
| Zeng | 2019 | | China | 164 | 110 | 54 | N/R | IHC | 47 | N/R | 7 |
| Zhang | 2019 | | US | 43 | 28 | 15 | N/R | IHC | 46 | N/R | 7 |
| Zhou | 2018 | | china | 136 | 91 | 45 | Protein | IHC | 54 | 45 | 8 |
| Zhu | 2018 | | china | 108 | 84 | 24 | Protein | IHC | 52 | N/R | 8 |

*Abbreviations: PDL1; programme death ligand-1 expression, IHC; immunohistochemistry ,QIF; quantitative immunofluorescence, RT-PCR; real time polymerase chain reaction, qPCR; quantative polymerase chain reaction, GS; genetic sequencing, NOS; Newcastle Ottawa scale, N/R; not reported.*

**Table S1.** Details regarding the 65 independent patient cohorts included in this systematic review. All studies were retrospective cohorts with level III evidence except Wei et al where this is not reported.
