# Supplementary material for: Clinicopathological and prognostic significance of programmed cell death ligand 1 expression in patients diagnosed with breast cancer: meta-analysis
Source: Br J Surg. 2021 May 8;108(6):622–31. doi: 10.1093/bjs/znab103 (PMC10364926; doi:10.1093/bjs/znab103)
Supplement: znab103_Supplementary_Data [file znab103_supplementary_data.zip › Fig. S2.docx]

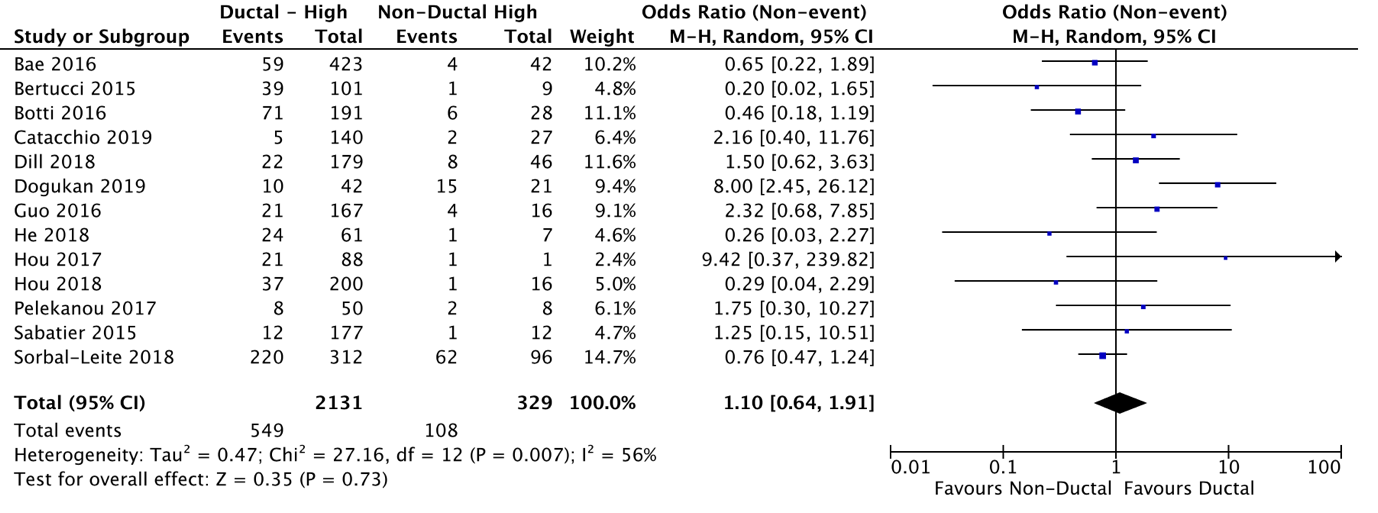


A


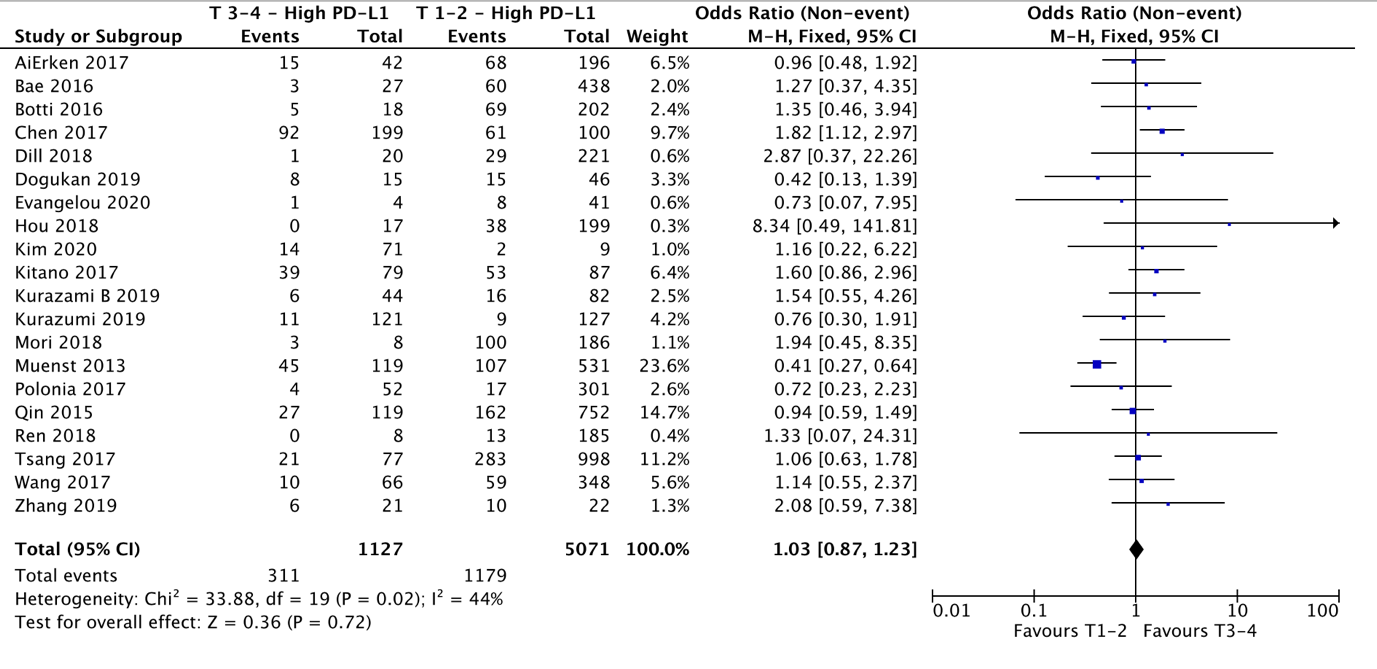


B


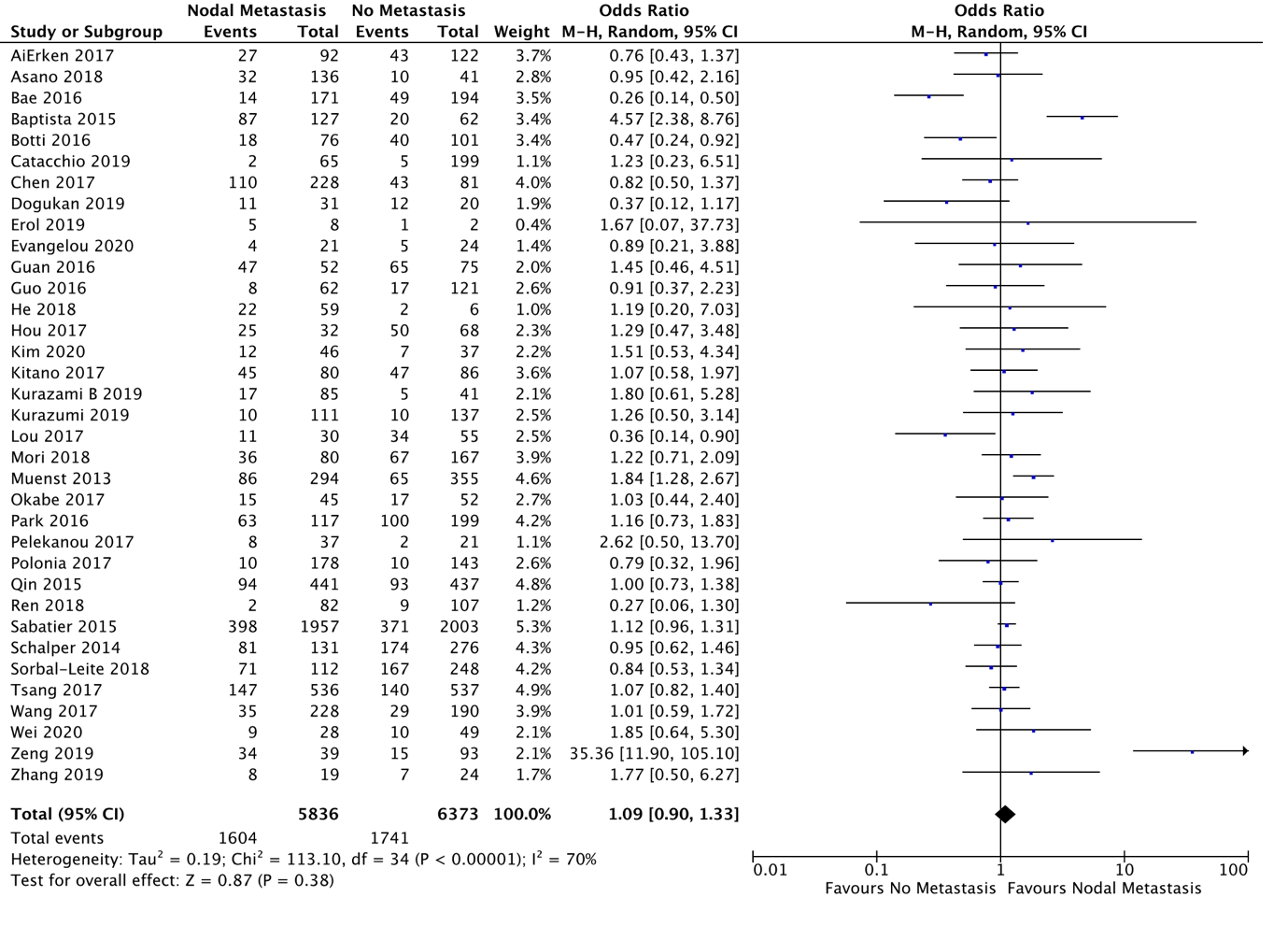


C


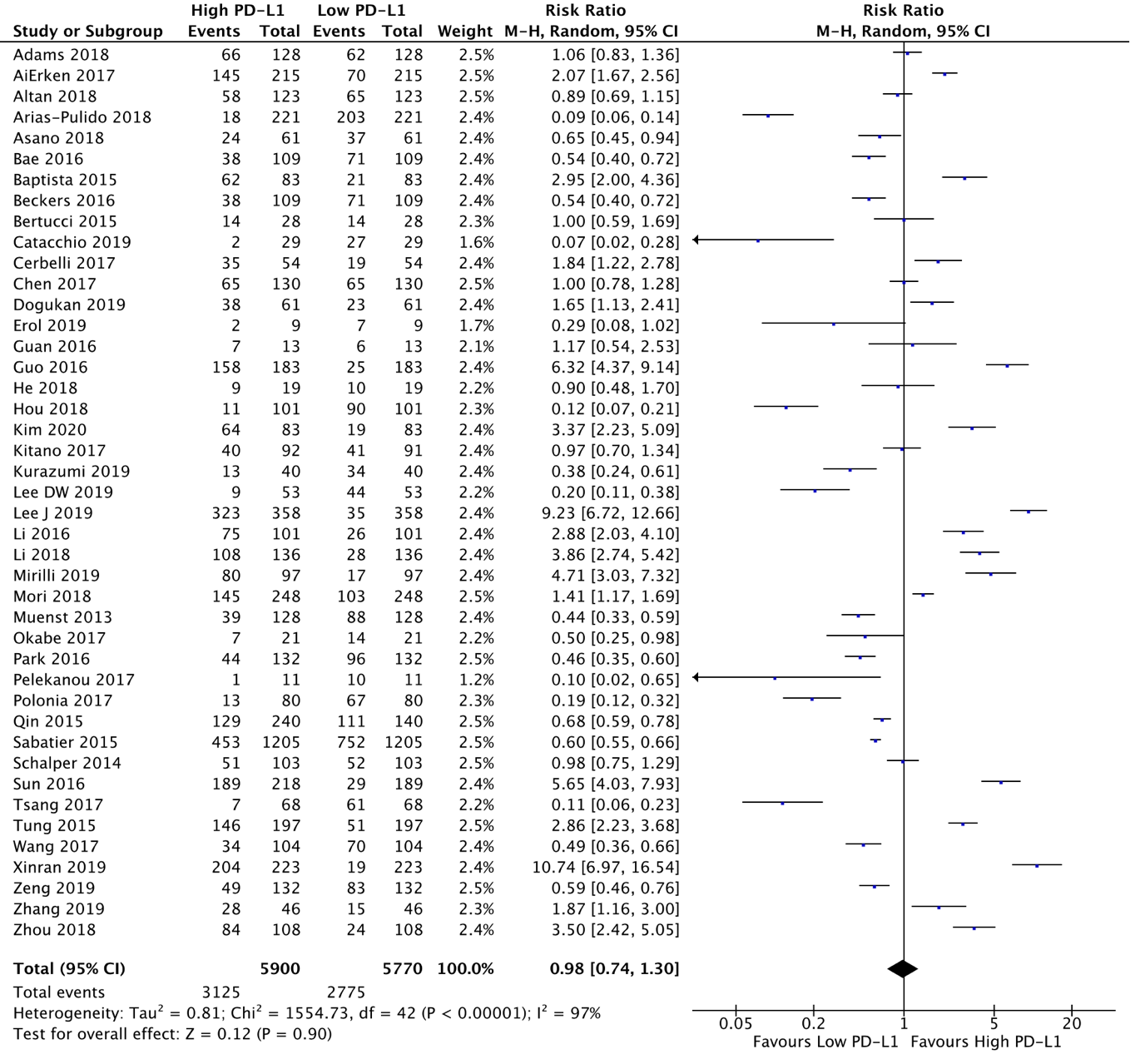


D


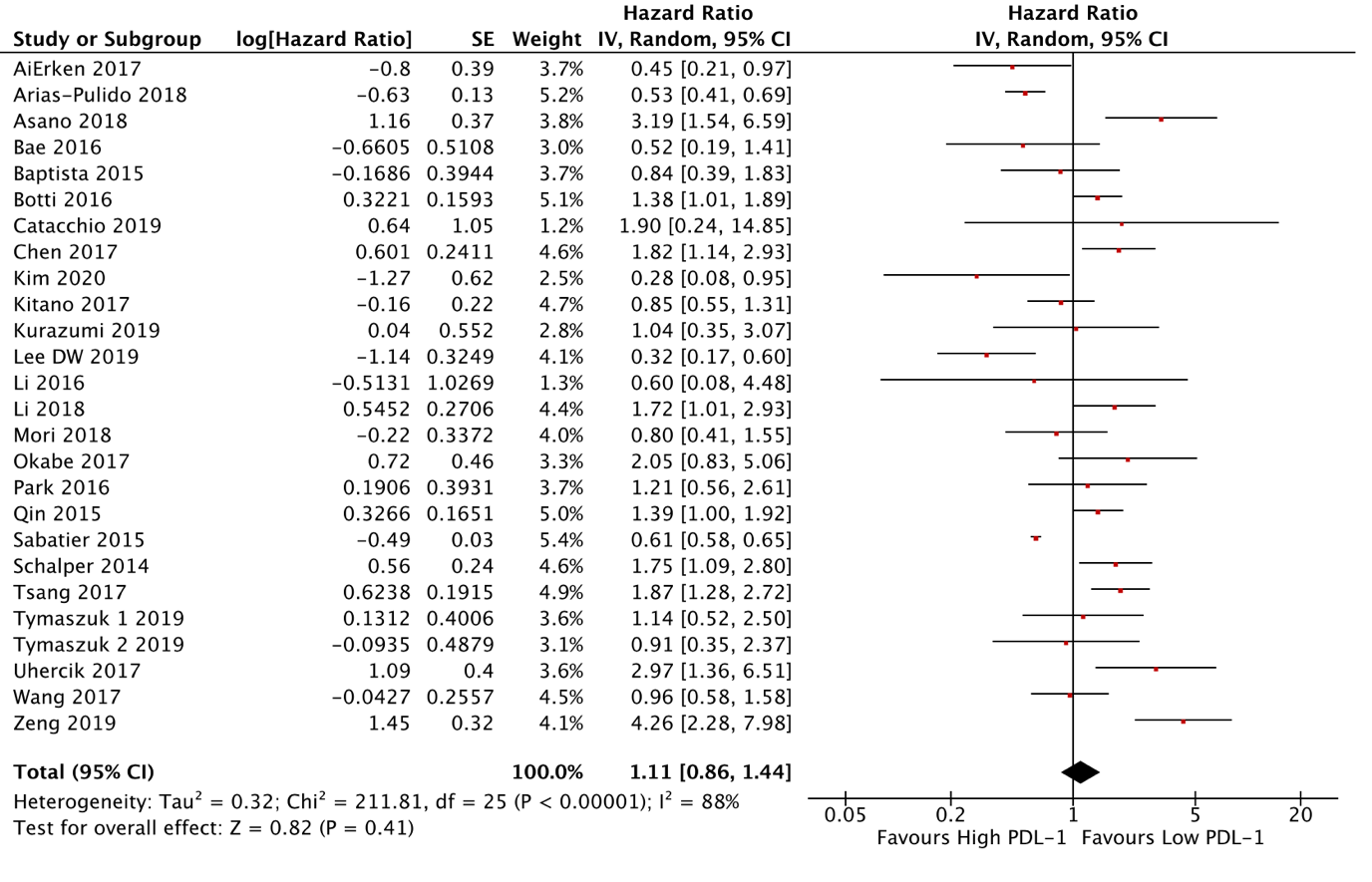


E


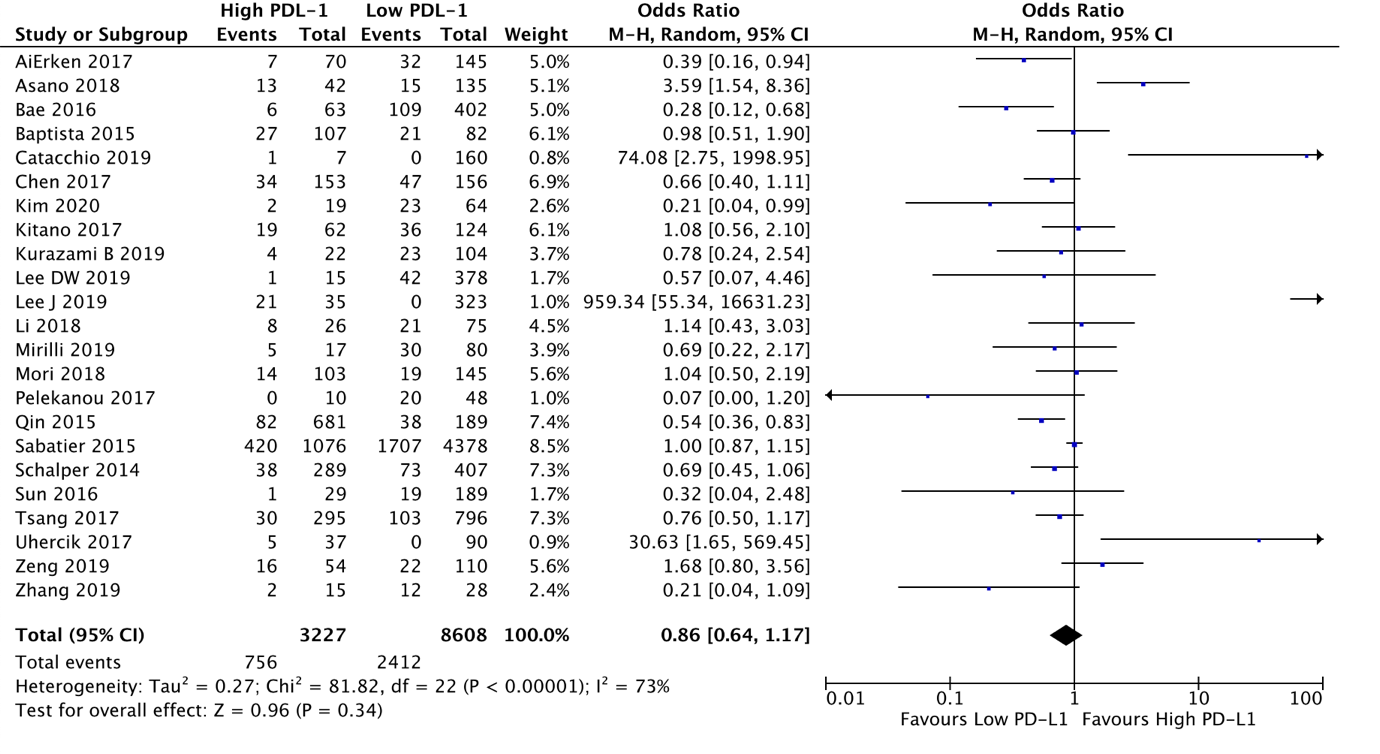


F


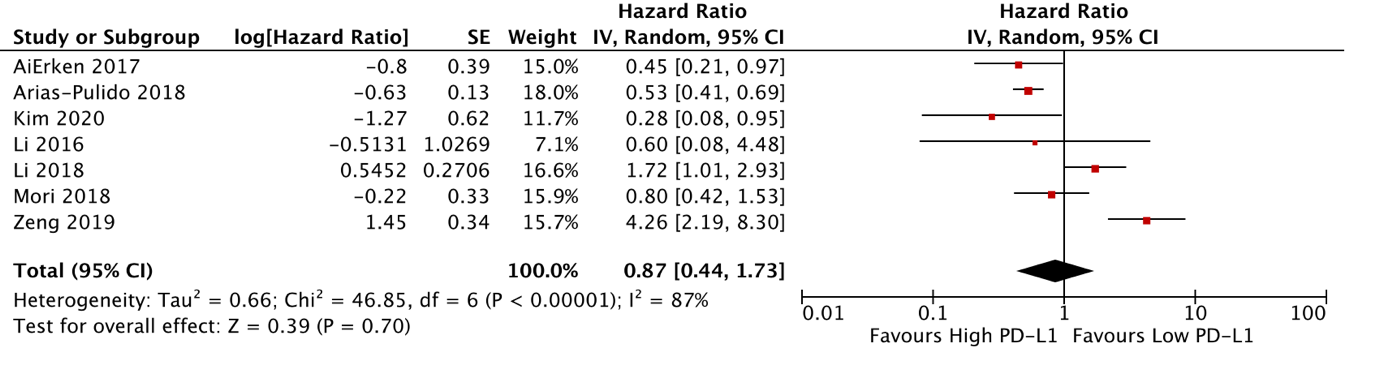


G


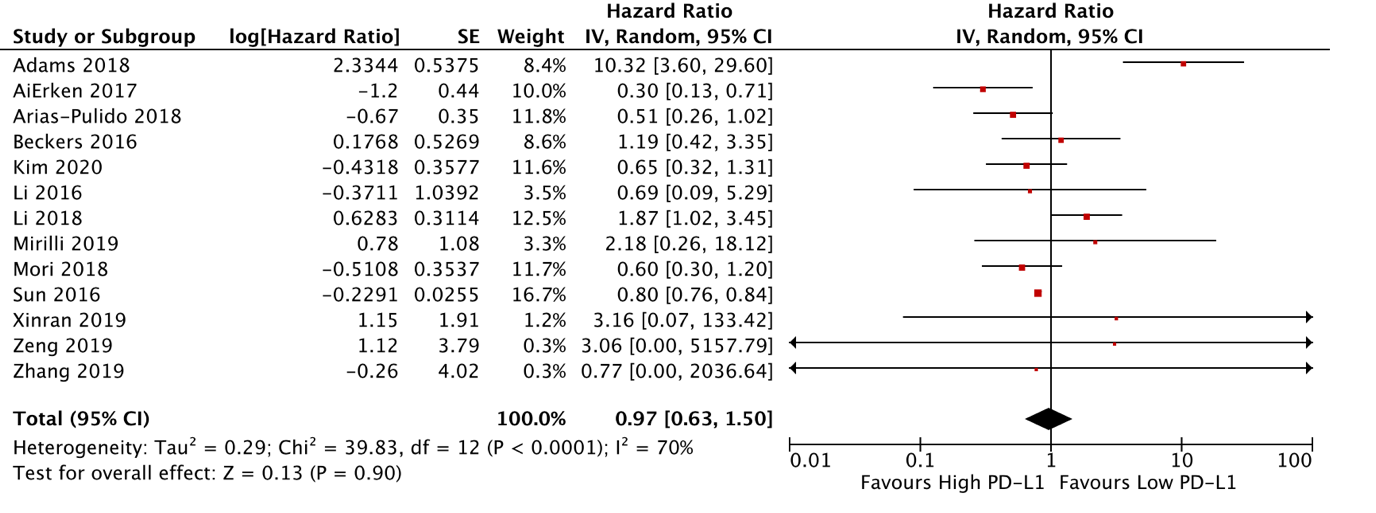


H

H

**Fig. S2:** Illustrating non-significant results comparing -high and -low programmed death ligand-1 expression with (A) invasive ductal carcinoma histopathological subtype, (B) pathological tumour staging, (C) pathological nodal metastasis, (D) triple negative molecular breast cancer molecular subtype, (E) disease-free survival, (F) disease recurrence at 5 years, (G) disease-free survival for patients diagnosed with triple negative breast cancer, and (H) overall survival for patients diagnosed with triple negative breast cancer.
