# Supplementary material for: Clinicopathological and prognostic significance of programmed cell death ligand 1 expression in patients diagnosed with breast cancer: meta-analysis
Source: Br J Surg. 2021 May 8;108(6):622–31. doi: 10.1093/bjs/znab103 (PMC10364926; doi:10.1093/bjs/znab103)
Supplement: znab103_Supplementary_Data [file znab103_supplementary_data.zip › Fig. S1.docx]

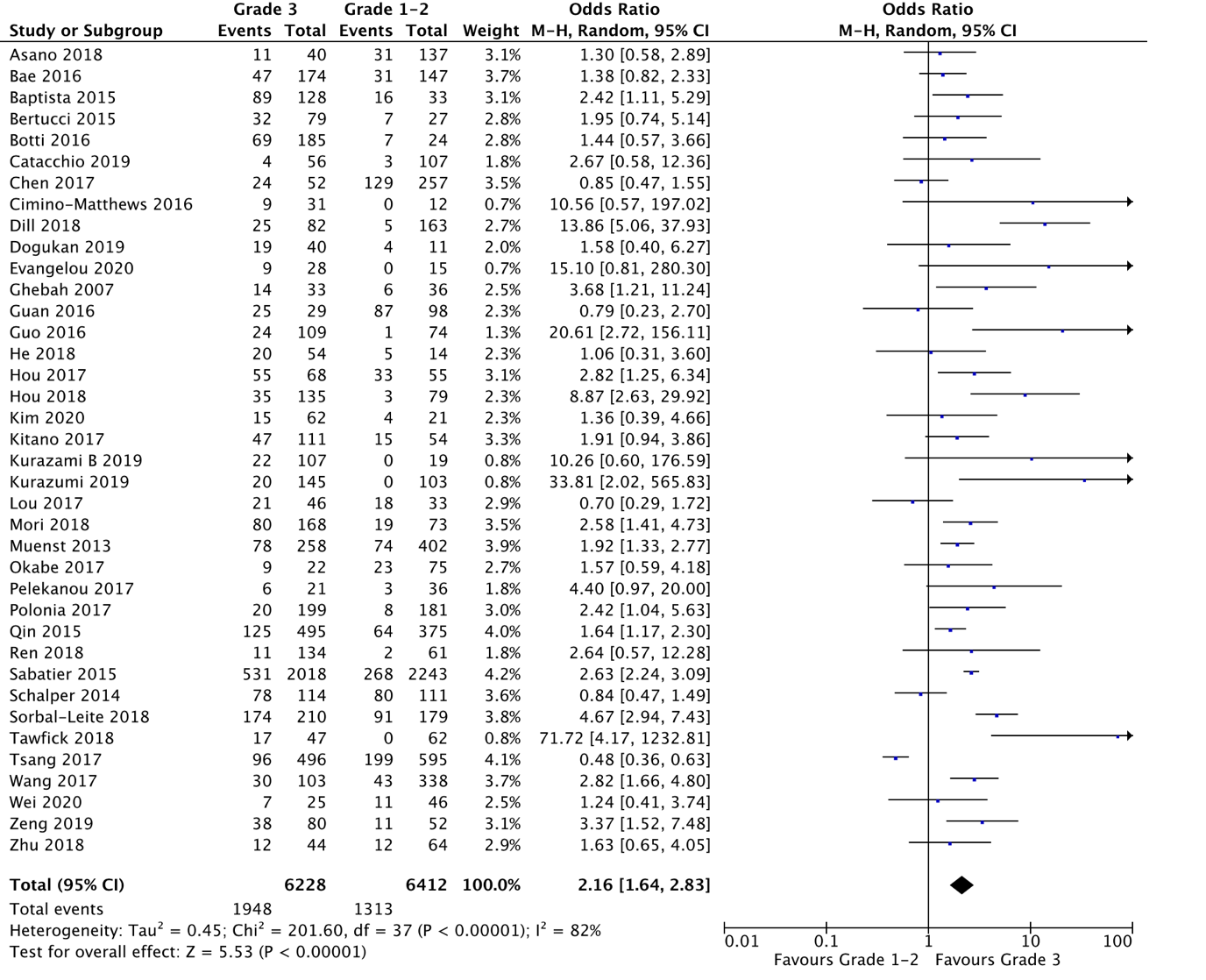


A


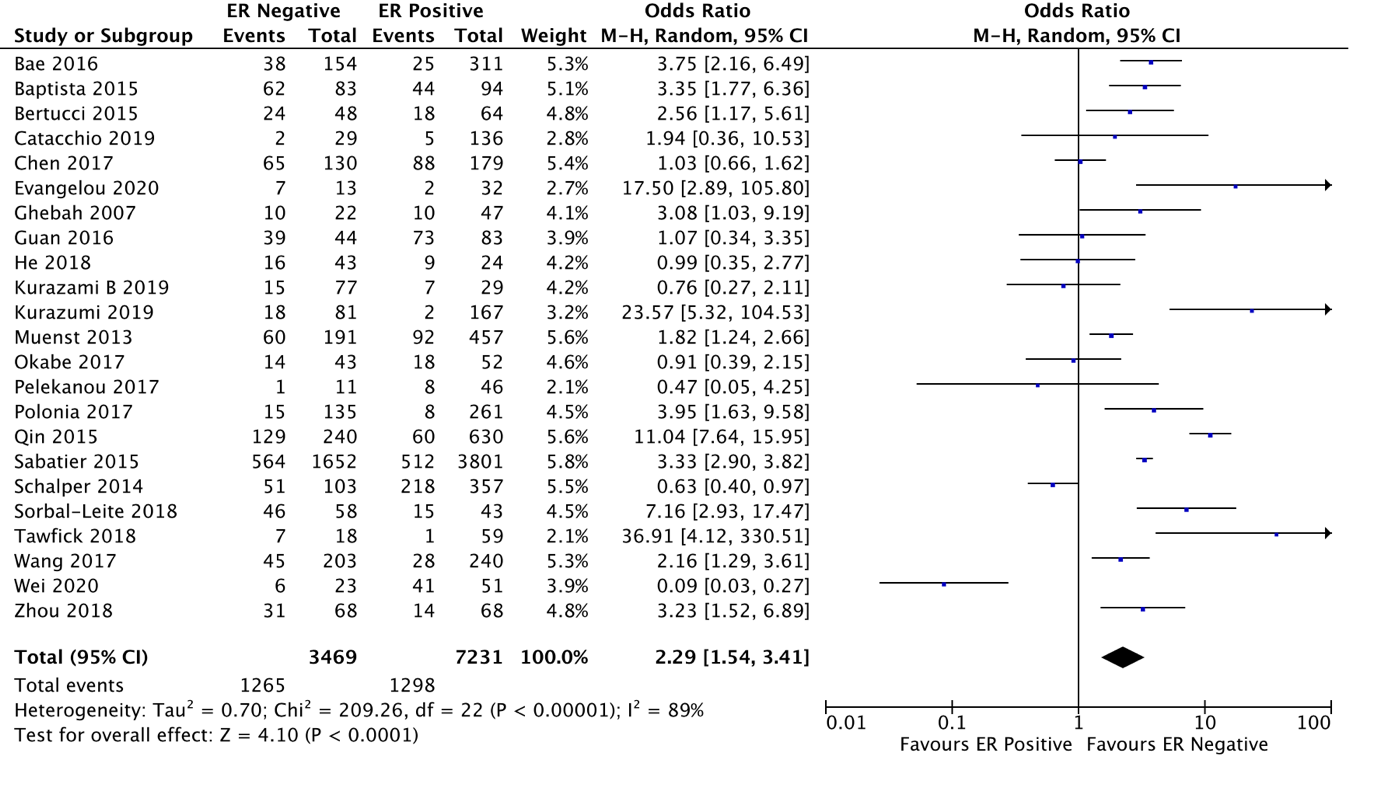


B


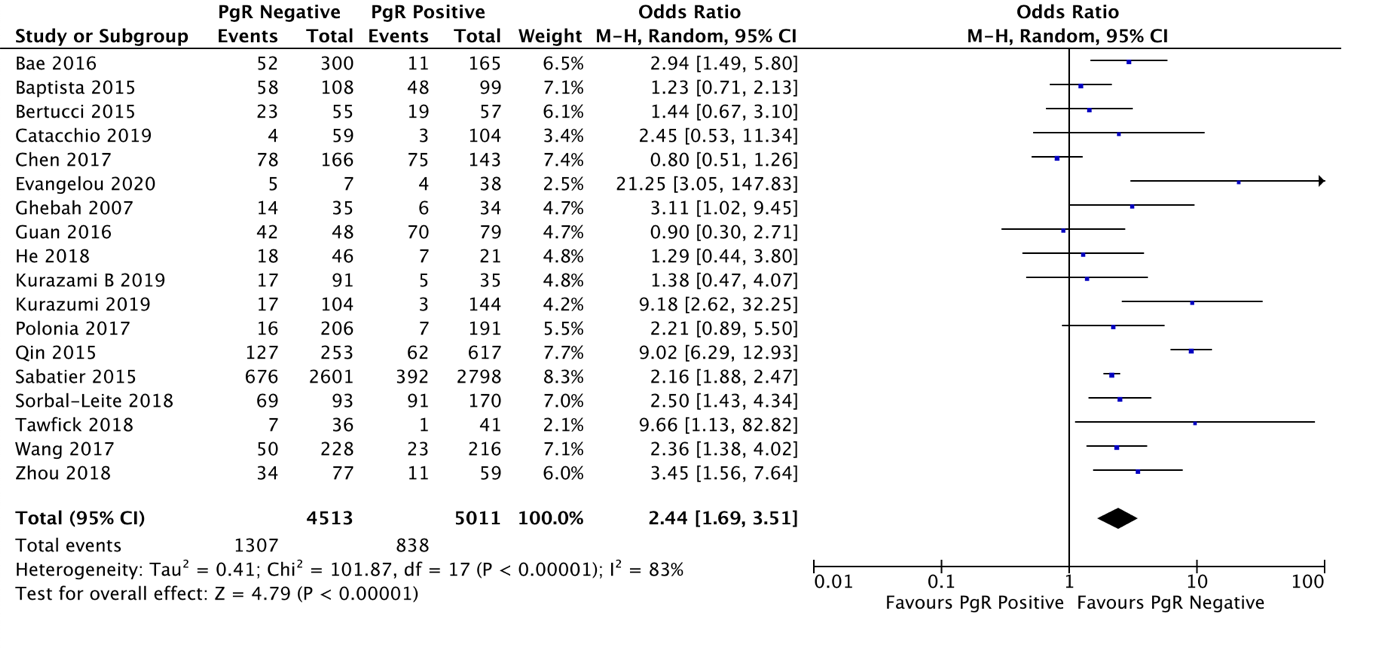


C


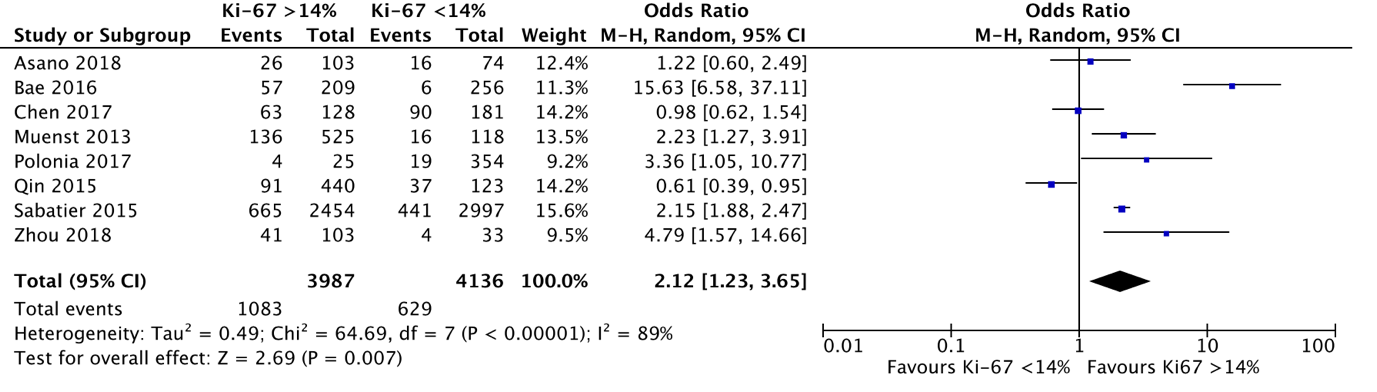


D

**Fig. S1:** Illustrating significant results comparing -high and -low programmed death ligand-1 expression with (A) histopathological tumour grade, (B) estrogen receptor status, (C) progesterone receptor status, and (D) Ki-67 proliferation indices greater than 14% and less than or equal to 14 in patients diagnosed with breast cancer.
